# Dysregulation of Catalase by a Sulphamoylated Estradiol Analogue Culminates in Antimitotic Activity and Cell Death Induction in Breast Cancer Cell Lines

**DOI:** 10.3390/molecules26030622

**Published:** 2021-01-25

**Authors:** Maphuti T. Lebelo, Anna M. Joubert, Michelle H. Visagie

**Affiliations:** Department of Physiology, University of Pretoria, Private Bag X323, Gezina, Pretoria 0031, South Africa; tebogo_lebelo@yahoo.com (M.T.L.); annie.joubert@up.ac.za (A.M.J.)

**Keywords:** ESE-one, superoxide dismutase, catalase, cell cycle progression, mitochondrial membrane depolarization

## Abstract

Recent findings revealed that 2-ethyl-17-oxoestra-1,3,5(10)-trien-3-yl sulfamate (ESE-one) induces antiproliferative activity and cell rounding dependent on the generation of superoxide anion, hydrogen peroxide and peroxyl radical. In the current study, the role of these reactive oxygen species was assessed in the activity exerted by ESE-one on cell cycle progression, mitochondrial membrane potential and cell death induction in breast tumorigenic cells. The influence of ESE-one was also investigated on superoxide dismutase and catalase activity. ESE-one induced a time-dependent accumulation of cells in the G_1_ phase and G_2_/M phase that is partially impaired by tiron and trolox and *N*,*N*′-dimethylthiourea suggesting that superoxide anion, hydrogen peroxide and peroxyl radical are required for these effects exerted by ESE-one. Flow cytometry data in MCF-7 cells demonstrated that tiron decreased depolarization of the membrane potential in ESE-one exposed cells, indicating that superoxide anion plays a role in the depolarization effects induced by ESE-one. Spectrophotometry data showed that ESE-one decreased catalase activity in both cell lines. This study contributes towards pertinent information regarding the effects of an in silico-designed sulfamoylated compound on antioxidant enzymes leading to aberrant quantities of specific reactive oxygen species resulting in antimitotic activity culminating in the induction of cell death in breast cancer cell lines.

## 1. Introduction

**Figure 1:**
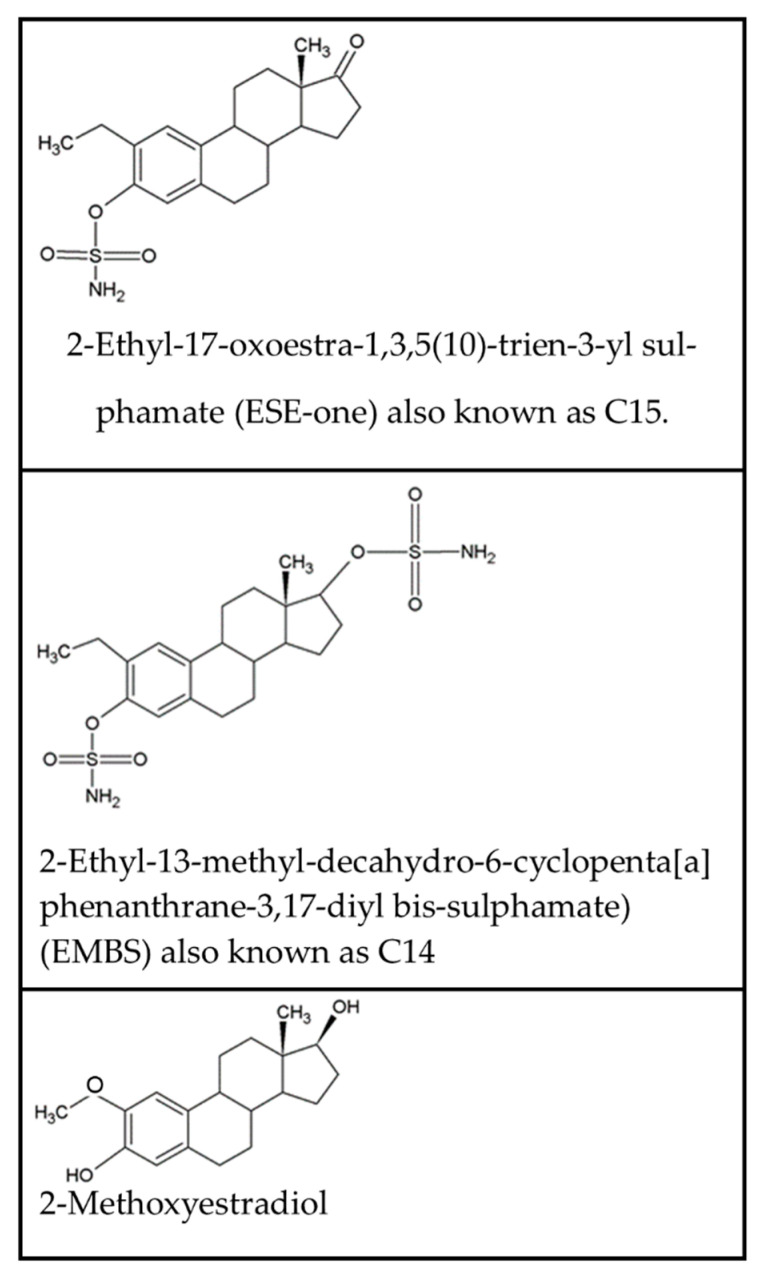
Chemical structure of 2-ethyl-17-oxoestra-1,3,5(10)-trien-3-yl sulfamate (ESE-one), 2-ethyl-13-methyl-decahydro-6-cyclopenta[a]phenanthrane-3,17-diyl bis-sulfamate (EMBS) and 2-methoxyestradiol.

The naturally occurring estradiol metabolite, 2-Methoxyestradiol (2ME), exerts anticancer and antiangiogenic activity [1]. However, 2ME possesses low bioavailability since it is degraded rapidly. The latter led to the in silico design of several estradiol analogs with increased potency including 2-ethyl-17-oxoestra-1,3,5(10)-trien-3-yl sulphamate (ESE-one) (Figure 1) [2].

Several previous studies have shown that sulfamoylated compounds such as 2-methoxyestradiol-bis-sulphamate and 2-ethyl-13-methyl-decahydro-6-cyclopenta[a]phenanthrane-3,17-diyl bis-sulfamate (EMBS) reduce cell growth and induce aberrant cell cycle activity in several tumorigenic cell lines including MCF-7 and MDA-MB-231 breast tumorigenic cell lines and the SNO esophageal tumorigenic cell line [2,3,4,5,6,7,8]. In addition, the antiproliferative and antimitotic activity exerted by EMBS in breast cancer cell lines was abrogated by N-acetyl cysteine (potent scavenger of reactive oxygen species) indicating that these effects induced by EMBS are dependent on increased reactive oxygen species generated due to exposure to EMBS [8]. Further studies demonstrated that exposure to sulfamoylated compounds, including ESE-one, resulted in decreased cell growth and increased quantities of hydrogen peroxide and superoxide when compared to their counterparts that was not sulfamoylated which had no significant effect. Further studies revealed that the chosen representative sulfamoylated compound, ESE-one, induced antiproliferative activity and cell rounding in an estrogen receptor (ER) positive adenocarcinoma breast cell line (MCF-7) and a metastatic triple negative breast cell line (MDA-MB-231). Furthermore, these effects exerted by ESE-one were inhibited by tiron, *N*,*N*’-dimethylthiourea (DMTU) and trolox indicating that the activity induced by ESE-one is dependent on the generation of superoxide anion, peroxyl radical and hydrogen peroxide. In addition, scavenging of hydroxyl radical, nitric oxide and singlet oxygen did not have any significant influence on the activity induced by ESE-one suggesting that these reactive oxygen species are not essential for the effects demonstrated by ESE-one [9]. However, the influence of ESE-one on the innate antioxidant system including superoxide dismutase (SOD) and catalase remains unknown. Furthermore, the role of reactive oxygen species is yet to be reported in the effects exerted by ESE-one on cell cycle progression, mitochondrial membrane potential and cell death induction.

Data obtained in the current study revealed pertinent information regarding the effects of a sulfamoylated compound on antioxidant enzymes resulting in aberrant quantities of specific reactive oxygen species resulting in antimitotic activity culminating in the induction of cell death in cancer cell lines. Identification of novel signaling pathways dependent on oxidative stress induced by antimitotic compounds to induce cell death in breast cell lines may potentially result in novel biochemical targets for improved future cancer treatment options.

## 2. Results

### 2.1. Cell Cycle Progression

**Figure 2:**
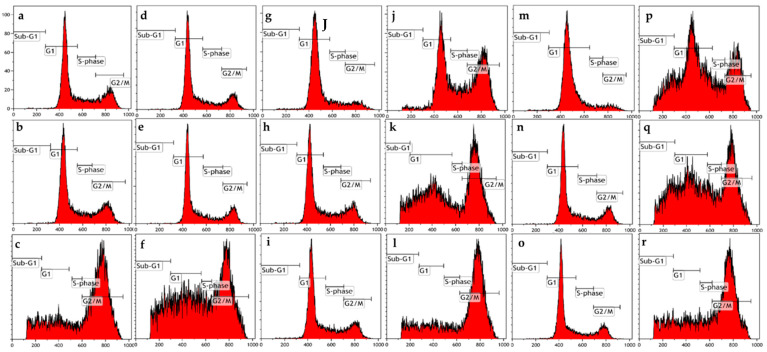
Cell cycle progression graphs of MCF-7 and MDA-MB-231 cells exposed to ESE-one in the presence or absence of several scavengers of reactive oxygen species (tiron, trolox and DMTU) for 24 h. ESE-one exposure resulted in a G_2_/M block in both MCF-7 and MDA-MB-231 cells. Tiron and DMTU exposure significantly decreased the number of cells blocked in sub-G_1_ phase in MDA-MB-231 cells and tiron as well as trolox exposure significantly decreased the number of cells blocked in G_2_/M phase in both MCF-7 and MDA-MB-231 cells. (**a**) MCF-7 cells cultured in complete growth medium, (**b**) vehicle-treated MCF-7 cells, (**c**) MCF-7 cells exposed to ESE-one only, (**d**) MDA-MB-231 cells cultured in complete growth medium, (**e**) vehicle-treated MDA-MB-231 cells, (**f**) MDA-MB-231 cells exposed to ESE-one, (**g**) MCF-7 cells exposed to tiron only, (**h**) MCF-7 cells exposed to trolox only, (**i**) MCF-7 cells exposed to DMTU only, (**j**) MCF-7 cells coexposed to tiron and ESE-one, (**k**) MCF-7 cells coexposed to trolox and ESE-one, (**l**) MCF-7 cells coexposed to DMTU and ESE-one, (**m**) MDA-MB-231 cells coexposed to tiron only, (**n**) MDA-MB-231 cells exposed to trolox only, (**o**) MDA-MB-231 cells exposed to DMTU only, (**p**) MDA-MB-231 cells coexposed to tiron and ESE-one, (**q**) MDA-MB-231 cells coexposed to trolox and ESE-one, (**r**) MDA-MB-231 cells coexposed to DMTU and ESE-one.

Propidium iodide, permeabilization using triton X-100 and ethanol fixation were used to investigate the effects of ESE-one for 24 h on cell cycle progression in the presence or absence of tiron (to investigate the role of superoxide anion), DMTU (to investigate the role of hydrogen peroxide) and trolox (to investigate the role of peroxyl radical) (Figure 2, Table 1 and Table 2). ESE-one exposure induced an accumulation of cells in the G_2_/M phase (48% and 59%) and a 16% and 25% increase of cells occupying the sub-G_1_ phase in MCF-7 and MDA-MB-231 cells, respectively. Tiron coexposure resulted in a decrease in the percentage of cells occupying the sub-G_1_ phase to 14% and 15%, and 17% and 42% G_2_/M phase in MCF-7 and MDA-MB-231 cell lines. Furthermore, ESE-one/trolox coexposure resulted in 24% and 28% of cells occupying the sub-G_1_ phase, and 24% and 43% percentage of cells occupying the G_2_/M phase in MCF-7 and MDA-MB-231 cells, respectively. Coexposure of ESE-one and DMTU demonstrated 11% and 16% percentage of cells occupying the sub-G_1_ phase, with 49% and 57% in the G_2_/M phase in MCF-7 and MDA-MB-231 cells, respectively. A significant decrease of cells was observed occupying the G_2_/M phase for combination exposure of ESE-one with tiron or trolox in MCF-7 and MDA-MB-231 cells when compared to cells treated with only ESE-one, and cells in sub-G1 in MDA-MB-231 due to tiron and DMTU. The latter indicates that peroxyl radical, superoxide anion and hydrogen peroxide is required for the effects exerted by ESE-one resulting in antimitotic activity.

**Figure 3:**
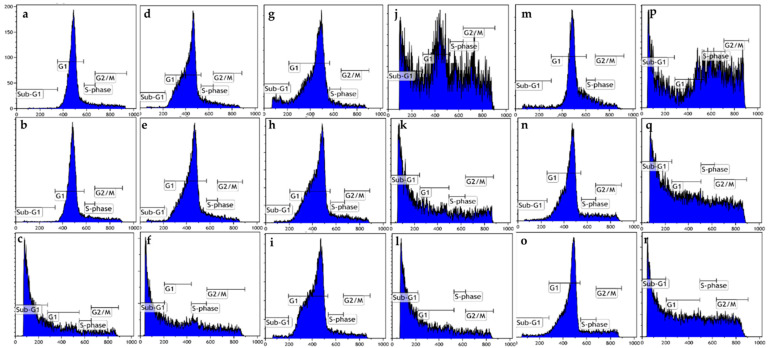
Cell cycle progression graphs of MCF-7 and MDA-MB-231 cells exposed to ESE-one in the presence or absence of various scavengers of reactive oxygen species (tiron, trolox and DMTU) for 48 h. ESE-one exposure resulted in a significant increase in the percentage of cells occupying the sub-G_1_ phase in both MCF-7 and MDA-MB-231 cells. Tiron, trolox and DMTU exposure significantly decreased the number of cells occupying the sub-G_1_ phase in MCF-7 cells and tiron as well as DMTU exposure significantly decreased the number of cells present in the sub-G_1_ phase in MDA-MB-231 cells. Tiron, trolox and DMTU significantly decreased the number of cells in the G_2_/M phase in MDA-MB-231 cells and only tiron had a significant effect in MCF-7 cells. (**a**) MCF-7 cells cultured in complete growth medium, (**b**) vehicle-treated MCF-7 cells, (**c**) MCF-7 cells exposed to ESE-one only, (**d**) MDA-MB-231 cells cultured in complete growth medium, (**e**) vehicle-treated MDA-MB-231 cells, (**f**) MDA-MB-231 cells exposed to ESE-one, (**g**) MCF-7 cells exposed to tiron only, (**h**) MCF-7 cells exposed to trolox only, (**i**) MCF-7 cells exposed to DMTU only, (**j**) MCF-7 cells coexposed to tiron and ESE-one, (**k**) MCF-7 cells coexposed to trolox and ESE-one, (**l**) MCF-7 cells coexposed to DMTU and ESE-one, (**m**) MDA-MB-231 cells exposed to tiron only, (**n**) MDA-MB-231 cells exposed to trolox only, (**o**) MDA-MB-231 cells exposed to DMTU only, (**p**) MDA-MB-231 cells coexposed to tiron and ESE-one, (**q**) MDA-MB-231 cells coexposed to trolox and ESE-one, (**r**) MDA-MB-231 cells coexposed to DMTU and ESE-one.

Cell cycle progression was also evaluated after exposure to ESE-one in the presence or absence of various scavengers of reactive oxygen species (tiron, DMTU and trolox) for 48 h (Figure 3, Table 3 and Table 4). Exposure to ESE-one only resulted in an increase of cells occupying the sub-G_1_ phase with 65% and 52% in MCF-7 and MDA-MB-231 cells, respectively. Coexposure with tiron resulted in 28% and 29% of cells occupying the sub-G_1_, and only 24% and 27% cells in the G_2_/M phase in MCF-7 and MDA-MB-231 cells, respectively. Trolox coexposure with ESE-one resulted in 55% and 40% cells occupying the sub-G_1_ phase and 14% and 23% cells occupying the G_2_/M phase in MCF-7 and MDA-MB-231 cells, respectively. DMTU coexposure resulted in 47% and 32% of cells present in the sub-G_1_ phase and 12% and 21% of cells occupying the G_2_/M phase in MCF-7 and MDA-MB-231 cells, respectively. Tiron had the greatest effective rescue effect in MCF-7 and MDA-MB-231 cells compared to the effects of exposure to trolox and DMTU in the presence of ESE-one. Trolox, tiron, and DMTU significantly decreased the number of cells in the sub-G_1_ phase. In MDA-MB-231 cells, tiron and DMTU significantly decreased the percentage of cells in the sub-G_1_ phase. Furthermore, the percentage of cells in the G_2_/M phase were significantly reduced by trolox, tiron and DMTU.

### 2.2. Mitochondrial Membrane Potential

**Figure 4:**
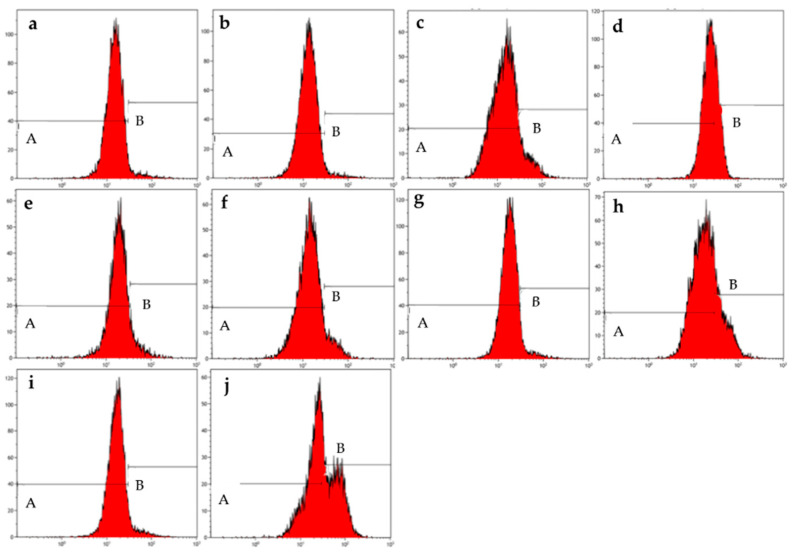
Mitochondrial membrane graphs of MCF-7 cells treated with ESE-one in the presence or absence of several scavengers of reactive oxygen species (tiron, trolox and DMTU). ESE-one only exposure resulted in depolarization of the mitochondrial membrane potential in MCF-7 cells and tiron countered that effect significantly. A represents polarized cells and B represents depolarized cells. Trolox and DMTU did not have an opposing effect on the membrane depolarization exerted by ESE-one (A indicates polarized population and B indicates depolarized population). (**a**) Cells cultured in complete growth medium, (**b**) vehicle-treated cells, (**c**) cells exposed to ESE-one, (**d**) cells exposed to carbonyl cyanide 3-chlorophenylhydrazone (CCCP), (**e**) cells exposed to tiron only, (**f**) cells coexposed to tiron and ESE-one, (**g**) cells exposed to trolox only, (**h**) cells coexposed to trolox and ESE-one, (**i**) cells exposed to DMTU only, (**j**) cells coexposed to DMTU and ESE-one.

**Figure 5:**
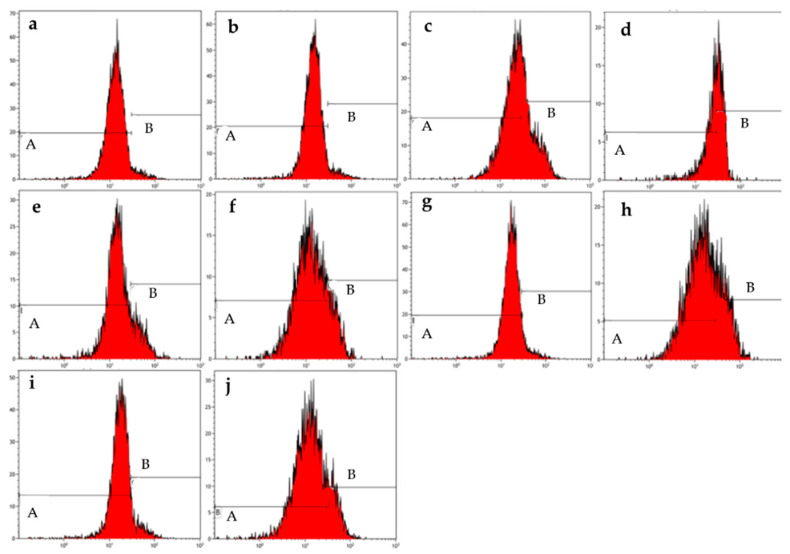
Mitochondrial membrane graphs of MDA-MB-231 cells exposed to ESE-one in the presence or absence of several scavengers of reactive oxygen species (tiron, trolox and DMTU). ESE-one only exposure resulted in depolarization of the mitochondrial membrane potential in MDA-MB-231 cells and tiron exposure resulted in an insignificant decrease in membrane depolarization. A represents polarized cells and B represents depolarized cells. Trolox and DMTU did not have an opposing effect on the membrane depolarization exerted by ESE-one (A indicates polarized population and B indicates depolarized population). (**a**) Cells cultured in complete growth medium, (**b**) vehicle-treated cells, (**c**) cells exposed to ESE-one, (**d**) cells exposed to carbonyl cyanide 3-chlorophenylhydrazone (CCCP), (**e**) cells exposed to tiron only, (**f**) cells coexposed to tiron and ESE-one, (**g**) cells exposed to trolox only, (**h**) cells coexposed to trolox and ESE-one, (**i**) cells exposed to DMTU only, (**j**) cells coexposed to DMTU and ESE-one.

The depolarization of the mitochondrial membrane potential was investigated by means of the MitoProbe JC-1 assay kit and flow cytometry in MCF-7 (Figure 4 and Table 5) and MDA-MB-231 (Figure 5 and Table 6) cells that were exposed to ESE-one and the presence or absence of trolox, tiron, or DMTU. This was done to assess the depolarization of the mitochondrial membrane potential induced by ESE-one and whether the coexposure with various scavengers of reactive oxygen species will counter the influence exerted by ESE-one on the mitochondrial membrane potential. When the mitochondria is intact, the membrane potential is polarized and thus the JC-1 dye will fluoresce red due to polarization. Damage to the mitochondria results in depolarization of the membrane potential and this will show green fluorescence of the JC-1 dye [10].

ESE-one only exposure resulted in 15% and 24% depolarization of the mitochondrial membrane potential in MCF-7 and MDA-MB-231 cells, respectively, indicating that ESE-one had deleterious effects on the mitochondria. Coexposure of ESE-one-treated cells with tiron significantly decreased the membrane depolarization to 9% whereas coexposure of ESE-one-treated cells with trolox or DMTU triggered an insignificant increase in the membrane potential depolarization (19% and 16%, respectively) in MCF-7 cells. However, in MDA-MB-231 cells, tiron (22%) and trolox (29%) coexposure with ESE-one induced an insignificant decrease in membrane depolarization (Figure 5 and Table 6 whereas DMTU (41%) coexposure with ESE-one led to a significant increase in mitochondrial membrane depolarization. Data thus indicates that superoxide anion might participate in the effect of ESE-one on the mitochondrial membrane potential in MCF-7 cells as tiron significantly decreased membrane potential depolarization in ESE-one exposed cells. This is the first study to report the effects of the various scavengers of specific reactive oxygen species on the activity of a sulfamoylated compound on mitochondrial membrane potential. Data obtained from this study indicated that DMTU had no inhibitory effect on the effects exerted by ESE-one in both cell lines. Furthermore, the observation that ESE-one in combination with DMTU induced a higher percentage of cells to become depolarized was observed, however, the reason for this is unknown at this stage and requires more investigation.

### 2.3. Antioxidant Activity

#### 2.3.1. Superoxide Dismutase Inhibition

**Figure 6:**
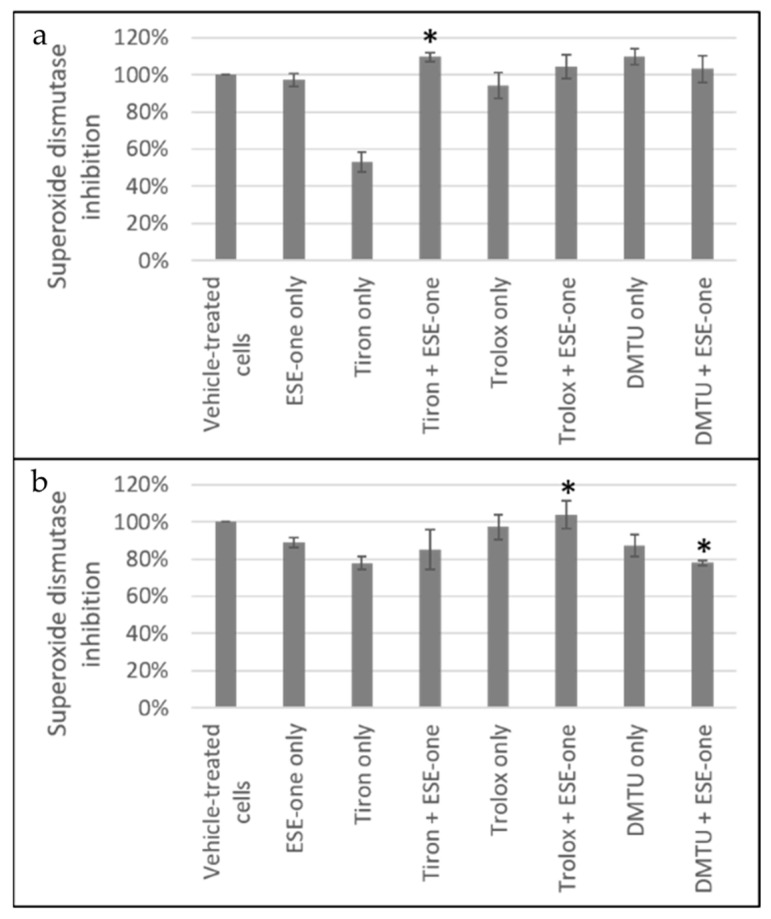
SOD inhibition graphs of (**a**) MCF-7 and (**b**) MDA-MB-231 cells exposed to ESE-one in the presence or absence of scavengers of reactive oxygen species (tiron, trolox and DMTU). Tiron, trolox and DMTU coexposure with ESE-one resulted in an increased SOD inhibition percentage compared to ESE-one only exposure in MCF-7 cells suggesting that increased generation of reactive oxygen species due to ESE-one exposure is not required for the inhibition of SOD. In MDA-MB-231, DMTU demonstrated a decrease in SOD inhibition percentage suggesting that hydrogen peroxide affects the superoxide anion activity. (**a**) MCF-7, (**b**) MDA-MB-231. An asterisk (*) indicates *p*-value (*p* < 0.05) compared to ESE-one treated cells.

Superoxide dismutase (SOD) inhibition activity was measured using a SOD activity kit with spectrophotometry in cells exposed to ESE-one in the presence or absence of various scavengers of reactive oxygen species. ESE-one only exposure demonstrated a 97% inhibition rate in MCF-7 cells and tiron coexposure resulted in 110%, trolox coexposure resulted in 104% and DMTU coexposure resulted in 103% SOD inhibition (Figure 6). This suggests that coexposure of ESE-one-treated cells with tiron, trolox or DMTU causes an increase in SOD inhibition however, these are insignificant. Cells exposed to only tiron demonstrated a significant decrease in SOD inhibition compared to vehicle-treated cells. In MDA-MB-231 cells, ESE-one exposure resulted in 89% SOD inhibition whereas coexposure with tiron, trolox and DMTU resulted in 85%, 104% and 78% SOD inhibition, respectively (Figure 6). These results demonstrate that DMTU has the best inhibitory effect on SOD compared to tiron and trolox.

#### 2.3.2. Catalase Activity

**Figure 7:**
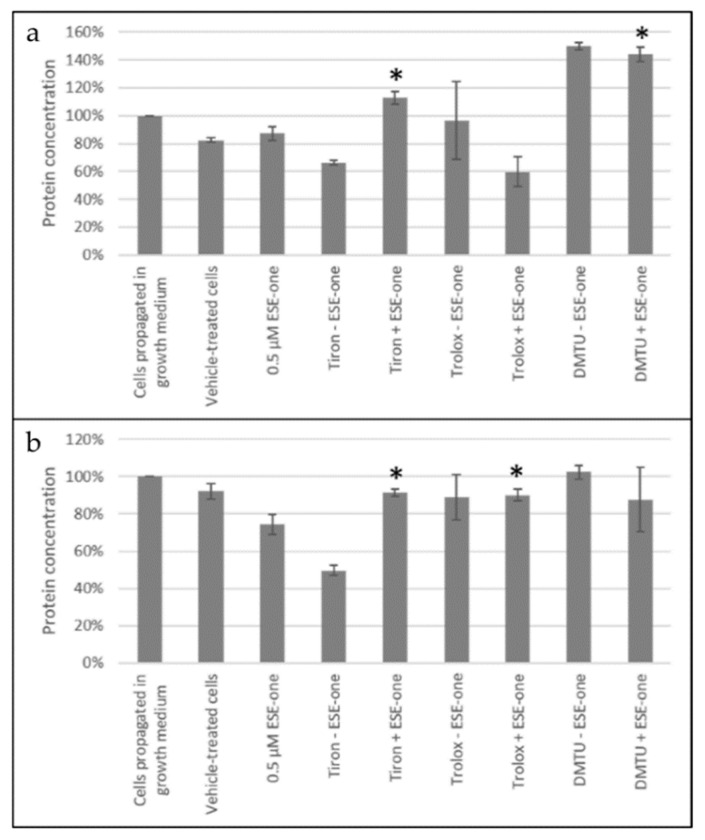
Catalase activity graphs of MCF-7 and MDA-MB-231 cells exposed to ESE-one in the presence or absence of scavengers of reactive oxygen species (tiron, trolox and DMTU). Tiron and DMTU coexposure with ESE-one increased the catalase protein concentration significantly in MCF-7 cells and in MDA-MB-231 cells, tiron and trolox induced a significant increase in catalase protein concentration. (**a**) MCF-7, (**b**) MDA-MB-231. An asterisk (*) indicates *p*-value (*p* < 0.05) compared to ESE-one treated cells.

Enzyme linked immunosorbent assay was used to study the catalase activity in cells exposed to ESE-one in the presence or absence of various scavengers of reactive oxygen species. Exposure to only ESE-one decreased catalase protein to 87% in MCF-7 cells compared to cells cultured in complete growth medium. However, coexposure with ESE-one and tiron and DMTU increased catalase concentration significantly to 113% and 144% when compared to ESE-one only exposed cells (Figure 7). Trolox, however, demonstrated a significant decrease in catalase protein concentration. These results demonstrate a significant increase due to tiron and DMTU exposure (combined with ESE-one) suggesting that the superoxide anion and hydrogen peroxide pathway are utilized by ESE-one. In MDA-MB-231 cells, ESE-one exposure resulted in decreased catalase protein to 74% compared to cells cultured in complete growth medium. However, coexposure with ESE-one and tiron and trolox increased catalase concentration significantly to 91% and 90%, respectively (Figure 7), however, DMTU had an insignificant increase in catalase protein concentration (88%).

## 3. Discussion

Sulfamoylated estradiol analogs have been explored in recent years and studies have reported that these derivatives exert antiproliferative and antimitotic activity resulting in cell death induction in various tumorigenic cell lines [2,3,4,5,6,7,8]. Recent studies have demonstrated that ESE-one possesses antiproliferative activity that is dependent on oxidative stress induced by increased peroxyl radical, superoxide anion and hydrogen peroxide [9]. However, the role of these reactive oxygen species on mitochondrial membrane potential and cell cycle progression remain unknown as well as the role of possible dysregulation of antioxidant enzymes by the sulfamoylated estradiol compound in breast tumorigenic cell lines.

Cell cycle progression data from the current study demonstrated an accumulation of cells in G_2_/M phase in both MCF-7 and MDA-MB-231 cells after exposure to ESE-one for 24 h which was opposed by tiron and trolox. However, exposure to ESE-one for 48 h resulted in a significant increase in cells occupying the sub-G_1_ phase which is indicative of cell death. This increase in the sub-G_1_ phase after exposure to ESE-one was inhibited by tiron, DMTU and trolox after 48 h in both MCF-7 and MDA-MB-231 cells. These effects were more prominent in the estrogen receptor breast adenocarcinoma epithelial MCF-7 cells compared to the estrogen receptor negative breast adenocarcinoma epithelial MDA-MB-231 cells. This indicates that superoxide anion, peroxyl radical and hydrogen peroxide play an essential role in the cell cycle disruption induced by ESE-one resulting in cell death (evident in the accumulation of cells in the sub-G_1_ phase). Previous studies have also reported that other sulfamoylated estradiol analogs (2-ethyloestradiol-3,17-*O*,*O*-*bis*-sulphamate, 2-ethyloestradiol-3-*O*-sulpfamate, 2-methoxyoestrone-3-*O*-sulpfamate and 2-methoxyoestrone-3-*O*-*O*-bis-sulfamate) induced a G_2_/M block in MDA-MB-231 cells [11]. In addition, exposure to diallyl trisulfide, a sulfamoylated antitumorigenic garlic extract, resulted in oxidative stress-dependent cell death in MCF-7 breast cancer cells and an accumulation of cells in sub-G_1_ which indicates cell death [12]. This is the first study to report that accumulation of cells in the sub-G1–and G2M phase is dependent on the generation of superoxide anion, peroxyl radical and hydrogen peroxide induced by the sulfamoylated estradiol ESE-one compound.

SOD and catalase are well-known endogenous antioxidant enzymes which inhibit superoxide anion and hydrogen peroxide, respectively [13,14]. The SOD inhibition rate was not distinctly affected by ESE-one and combination exposure with any of the scavengers of reactive oxygen species. However, ESE-one combination exposure with tiron and combination exposure with DMTU increased the catalase protein concentration in MCF-7 cells indicating that tiron and DMTU play a role in hydrogen peroxide inhibition. Tiron and trolox had a similar effect in MDA-MB-231 cells. An increase in catalase protein concentration indicated a decline in the hydrogen peroxide concentration suggesting that superoxide anion and peroxyl radical upstream of hydrogen peroxide push the reaction forward thus promoting the conversion of hydrogen peroxide to water and oxygen by catalase. Tiron is a well-known membrane-permeable scavenger of superoxide since it mimics SOD [15]. However, the mechanism of action responsible for increased SOD inhibition activity following coexposure with tiron and ESE-one in the MCF-7 cell line remains elusive and warrants further investigation since tiron abrogated SOD inhibition activity and ESE-one had no significant effect. Trolox is a water-soluble analog of vitamin E and a potent scavenger of the peroxyl radical, and the current study indicates that coexposure does restore SOD inhibition activity [16]. In addition, tiron also rescues the catalase concentration of cells coexposed with ESE-one in both cell lines since it mimics SOD [15]. The underlying differential cell line-dependent data obtained in the antioxidant activity assays after coexposure with the various scavengers and ESE-one remain yet to be reported and further research is required since it might be due to a variety of factors including receptor status or glycolytic ability. In addition, future research will include investigating the upstream mechanism of action leading to oxidative stress responsible for the dysregulated catalase activity resulting in cell cycle abnormalities, mitochondrial membrane depolarization and cell death. Reactive oxygen species are involved in the intrinsic pathway of apoptosis which involves mitochondrial permeabilization resulting in cytochrome *c* release [17,18]. This process is inhibited by antioxidants, including catalase, which oxidize the ROS. Mitochondrial SOD and catalase are regulated by protein kinase B (Akt)/Forkhead box (Foxo) transcription factor pathway however, FoxO3a specifically regulates catalase. FoxO3a is regulated by Akt signaling pathway; this signaling pathway is said to suppress catalase expression in cancer cells [19]. The data obtained in the studies demonstrating catalase activity suggested that ESE-one may induce cell death dependent on reactive oxygen species via the Akt signaling pathway since catalase protein was suppressed in ESE-one only treated cells which was recovered by cotreatment with tiron, trolox and DMTU. Reactive oxygen species are generated by the mitochondria and elevated quantities of reactive oxygen species can potentially be cytotoxic, but the mitochondrial antioxidants (SOD and catalase) eliminate the reactive oxygen species to maintain homeostasis thus it is important to assess the mitochondrial membrane potential [20,21,22].

Mitochondrial integrity is an important factor in apoptosis signaling: a drop in the mitochondrial membrane potential is indicative of apoptosis [23]. Elevated levels of reactive oxygen species cause disruptions in the mitochondria which results in opening of the mitochondrial channel and ultimately, a drop of mitochondrial membrane potential [24,25]. A previous study demonstrated the antiproliferative and antimitotic effects induced by EMBS (a sulfamoylated compound) including mitochondrial membrane damage in MDA-MB-231 cells which were inhibited by N-acetyl cysteine, these new findings suggested that sulfamoylated estradiol analogs induce mitochondrial damage via reactive oxygen species [8]. ESE-one exposure induced mitochondrial depolarization in MCF-7 and MDA-MB-231 cells which was rescued by tiron in MCF-7 cells. This suggests that superoxide anion plays a role in the depolarization of the mitochondrial membrane induced by ESE-one. In addition, the data obtained from this study indicates that DMTU had no inhibitory effect on the effects exerted by ESE-one in both cell lines. Furthermore, the observation that ESE-one in combination with DMTU induced a higher percentage of cells to become depolarized was observed, however, the reason for this is unknown at this stage and requires more investigation.

## 4. Materials and Methods

### 4.1. Cell Lines

The MDA-MB-231 cell line originated from a human adenocarcinoma metastatic site. The MDA-MB-231 cell line is a triple negative tumorigenic breast cell line referring to the absence of receptors for estrogen, progesterone or human epidermal growth factor [26]. The MCF-7 cell line also originated from an adenocarcinoma site; however, the MCF-7 cell line does express receptors for estrogen and progesterone. Both cell lines were purchased from the American Type Culture Collection (American Type Culture Collection, Manassas, VA, USA) [27]. Both tumorigenic cell lines were cultured in a humidified atmosphere at 37 °C and 5% CO_2_. Cells were cultured in complete growth medium consisting of Dulbecco’s minimum essential medium eagle (DMEM) supplemented with 100 U/mL penicillin G, 100 mg/mL streptomycin and 10% heat-inactivated fetal calf serum (56 °C, 30 min),

### 4.2. Reagents

All chemicals and reagents were purchased from Sigma Chemical Co (Sigma Chemical Co, St. Louis, MO, USA) unless stated otherwise. Propidium iodide, trolox, DMTU and tiron were obtained from Sigma Chemical Co. (Sigma Chemical Co., St. Louis, MO, USA). The superoxide dismutase activity assay kit (colorimetric) and human catalase activity kit SimpleStep were purchased from Abcam plc. (Cambridge, England, UK). Mitoprobe JC-1 assay kit was acquired from Thermo Fisher Scientific (Waltham, MA, USA).

All experiments were performed in the presence and absence of the reactive oxygen species scavengers including tiron that scavenges superoxide, trolox that scavenges the peroxyl radical and DMTU that scavenges hydrogen peroxide. Previous studies have been performed which indicated that these scavengers inhibited the antiproliferative activity induced by ESE-one in a dose-dependent manner. The concentrations of the scavengers used in this study are 5 mM tiron, 8 mM DMTU or 80 µM trolox since the previous experiments indicated that scavengers inhibited the antiproliferative activity exerted by ESE-one optimally at these doses [9].

### 4.3. Methods

#### 4.3.1. Cell Cycle Progression

Cell cycle progression was investigated by means of ethanol fixation, propidium iodide and flow cytometry. Propidium iodide stains the deoxyribonucleic acid (DNA) and thus permits for the quantification of DNA correlating with the different stages of the cell cycle [1].

Cells (MCF-7 and MDA-MB-231) were seeded at a density of 500,000 cells per T25 cm^2^ tissue culture flask and incubated at 37 °C and 5% CO_2_ for 24 h to allow for cell attachment. Cells were then exposed to ESE-one (0.5 μM) in the presence or absence of various scavengers of reactive oxygen species for 24 and 48 h in a humidified atmosphere at 37 °C and 5% CO_2_. Upon termination, cells were trypsinized, resuspended in 1 mL complete growth medium and then samples were centrifuged for 5 min at 300× *g*. The supernatant was removed, and the pellet was resuspended in ice-cold phosphate buffer saline (PBS) (200 μL) containing 0.1% fetal calf serum. Thereafter, 4 mL of 70% ice-cold ethanol was added to the samples in a drop-wise manner and samples were kept at 4 °C for longer than 24 h. Cells were then centrifuged at 300× *g* for 5 min, supernatant was removed, and the pellet was resuspended in 1 mL PBS containing propidium iodide (40 µg/mL), ribonuclease A (100 µg/mL) and triton X-100 (0.1%) and incubated at 37 °C for 45 min. Propidium iodide fluorescence was measured with the Gallios flow cytometer (Beckman Coulter, Inc. (Indianapilis, IN, USA). Data from cell debris including dust and particles smaller than apoptotic bodies were removed from further analysis in addition to the removal of cellular clumps involving 2 cells or more. Cell cycle distributions were quantified with Kaluza analysis software version 2.0 software from Beckman Coulter Life Sciences (Indianapolis, IN, USA) by assigning relative DNA content per cell to sub-G_1_, G_1_, S and G_2_/M fractions by means of Kaluza analysis software version 2.0 software from Beckman Coulter Life Sciences (Indianapolis, IN, USA).

#### 4.3.2. Mitochondrial Membrane Potential

The influence of ESE-one on the cells’ mitochondrial potential was investigated using MitoProbe JC-1 Assay Kit employing flow cytometry. JC-1 dye fluoresces green if the mitochondrial potential is depolarized and fluoresces red when the mitochondrial membrane potential is polarized. Depolarization of the mitochondrial membrane is suggestive that the mitochondrial apoptotic pathway is induced. The green fluorescence was measured at 525 nm excitation whereas the red fluorescence was measured at 575 nm excitation for JC-1 dye [28,29,30].

Cells (MCF-7 and MDA-MB-231) were seeded at a density of 500,000 cells per 25 cm^2^ tissue flask and incubated at 37 °C and 5% CO_2_ for 24 h to allow for attachment. Cells were then exposed to ESE-one (0.5 μM) in the presence or absence of various scavengers of reactive oxygen species for 24 h in a humidified atmosphere at 37 °C and 5% CO_2_. Thereafter, cells were trypsinized and resuspended in 1 mL warm PBS. Samples were then centrifuged at 300× *g* for 5 min and the supernatant was discarded afterwards. CCCP (50 μM) was added to the positive control sample and incubated for 5 min at 37 °C and 5% CO_2_ as instructed by the manufactures. Samples were then centrifuged, and the supernatant was discarded. The pellet was then resuspended in warm PBS (1 mL) and subsequently JC-1 dye solution (2 µM) was added to each sample. Samples were then incubated for 15 min at 37 °C and 5% CO_2_. After the incubation period, cells were centrifuged at 300× *g* for 5 min and the supernatant was discarded. Cells were then resuspended in warm PBS (1 mL) and centrifuged at 300× *g* for 5 min and the supernatant was discarded. PBS (0.5 mL) was added to each sample and samples were processed using the Gallios flow cytometer (Beckman Coulter, Inc. (Indianapolis, IN, USA) at an excitation wavelength of 488 nm. Mitochondrial membrane potential data was analyzed using Kaluza analysis software version 2.0 software from Beckman Coulter Life Sciences (Indianapolis, IN, USA) by quantifying the mitochondrial depolarization. The percentage of cells were calculated that represented polarized and depolarized cells within the histogram relative to the histogram of cells propagated in complete growth medium using the statistics provided by Kaluza analysis software version 2.0 software from Beckman Coulter Life Sciences (Indianapolis, IN, USA).

#### 4.3.3. Antioxidant Activity

##### Superoxide Dismutase Activity (Colorimetric Assay)

The influence of ESE-one on the cells’ antioxidant systems was investigated by quantifying SOD using the superoxide dismutase activity assay kit obtained from Abcam plc (Cambridge, England, UK). Quantification of SOD was used as an indication of the influence of ESE-one on the cells’ innate antioxidant defense systems [31]. The superoxide dismutase activity assay kit utilizes a water-soluble tetrazolium salt, WST-1, that generates a formazan dye upon reduction with superoxide anion. The rate of WST-1 reduction is linearly related to the inhibition activity of xanthine oxidase by SOD [32]. SOD is an antioxidant enzyme involved in the defense system against ROS. SOD catalyzes the reaction of superoxide radical anion to hydrogen peroxide [31].

Cells (MCF-7 and MDA-MB-231) were seeded at a density of 2,000,000 cells per 75 cm^2^ tissue culture flask and incubated at 37 °C and 5% CO_2_ for 24 h to allow for attachment. Cells were then exposed to ESE-one (0.5 μM) in the presence or absence of various scavengers of reactive oxygen species for 24 h in a humidified atmosphere at 37 °C and 5% CO_2_. Cells were trypsinized and samples were placed in ice-cold 0.1 M Tris/HCl (pH 7.4) containing 0.5% triton X-100, 5 mM β-mercaptoethanol and 0.1 mg/mL phenylmethylsulfonyl fluoride. Cells were centrifuged at 14,000× *g* for 5 min at 4 °C. The supernatant was transferred to new eppendorfs and kept on ice. Supernatant (10 µL) was then transferred to the 96-well plate (row 1 and row 3) and double distilled water (10 µL) was added to row 2 and row 4. A further WST working solution (100 µL) was added to all the wells and SOD enzyme solution (10 µL) was only added to row 1 and 2. SOD dilution buffer (10 µL) was added to row 3 and row 4 thereafter, incubated for 1 h on the plate shaker at 400 rpm (covered in foil). After the incubation period, the absorbance of the samples was determined at 450 nm using an EPOCH Microplate Reader (Biotek Instruments, Inc. (Winooski, VT, USA). Data was then exported and evaluated using Microsoft Excel 2010 (Microsoft Corporation, WA, USA).

##### Catalase Activity (Enzyme Linked Immunosorbent Assay)

The influence of ESE-one on the cells’ innate antioxidant systems was investigated by quantifying catalase by means of the human catalase activity kit SimpleStep purchased from Abcam plc (Cambridge, England, UK). Hydrogen peroxide is catalyzed to water and oxygen by catalase and thus protecting the cell from oxidative stress [33].

Cells (MCF-7 and MDA-MB-231) were seeded at a density of 2,000,000 cells per 75 cm^2^ tissue culture flask and incubated at 37 °C and 5% CO_2_ for 24 h to allow for attachment. Cells were then exposed to ESE-one (0.5 μM) in the presence or absence of various scavengers of reactive oxygen species for 24 h in a humidified atmosphere at 37 °C and 5% CO_2_. Cells were then scraped off the surface of the flask and suspended in PBS. After centrifuging, the supernatant was discarded, and ice-cold cell extraction buffer (100 µL) was added to each sample and left on ice for 20 min. Thereafter, cells were centrifuged at 14,000× *g* for 15 min and the supernatant (50 µL) was transferred to a 96-well plate. A further antibody cocktail (50 µL) was added to the wells (providing a final volume of 100 µL) and incubated on the plate shaker for 90 min at 400 rpm. Wells were then washed thrice with wash buffer (250 µL) and TMB substrate (100 µL) was added to the wells and incubated for a further 15 min on the plate shaker at 400 rpm. After the incubation time, stop solution was added to the wells and read on the spectrophotometry. The absorbance of the samples was determined at 450 nm using an EPOCH Microplate Reader (Biotek Instruments, Inc. (Winooski, VT, USA). Data was then exported and evaluated using Microsoft Excel 2010 (Microsoft Corporation, WA, USA).

### 4.4. Statistics

Quantitative data was obtained from spectrophotometry (antioxidant SOD and catalase activity) and flow cytometry (cell cycle progression and mitochondrial membrane potential). Three independent repeats were performed for all experiments where the averages and the standard deviations were determined. Averages are demonstrated using bar charts and standard deviations are displayed with errors bars. A *p*-value < 0.05 calculated by means of the Student *t-*test was utilized for statistical significance and is specified using an asterisk (*). Flow cytometry analysis includes at least 10,000 events. The percentage of cells were calculated that represented polarized and depolarized cells within the histogram relative to the histogram of cells propagated in complete growth medium using the statistics provided by Kaluza analysis software version 2.0 software from Beckman Coulter Life Sciences (Indianapolis, IN, USA). Cell cycle distributions were quantified with Kaluza analysis software version 2.0 software from Beckman Coulter Life Sciences (Indianapolis, IN, USA) by assigning relative DNA content per cell to sub-G_1_, G_1_, S and G_2_/M fractions using Kaluza analysis software version 2.0 software from Beckman Coulter Life Sciences (Indianapolis, IN, USA).

## 5. Conclusions

Previous research has indicated that sulfamoylated estradiol compounds exert antiproliferative and antimitotic activity in several tumorigenic cell lines [7]. Recent findings reported that ESE-one, a representative sulfamoylated estradiol analog, reduces cell growth and induces cell rounding by means of increased quantities of superoxide anion, peroxyl radical and hydrogen peroxide [9]. The current study suggests that ESE-one induces a time-dependent accumulation of cells in the G_2_/M and G_1_ phase that is partially impaired by tiron and trolox and DMTU suggesting that superoxide anion, hydrogen peroxide and peroxyl radical is required for the effects exerted by ESE-one resulting in antimitotic activity and induction of cell death. Furthermore, mitochondrial membrane depolarization studies in MCF-7 cells demonstrated that tiron significantly decreased depolarization of the membrane potential in ESE-one exposed cells, indicating that superoxide anion plays a role in the depolarization effects exerted by ESE-one on the mitochondrial membrane potential. Data relating to catalase activity showed that ESE-one dysregulated catalase activity in both cell lines. Research contributing towards the role of reactive oxygen species in antimitotic activity and cell death induction in cancer cells may lead to optimized future mechanistic and pharmacogenomic models for successful oxidative stress-dependent targeting and thereby improve current therapy targeting ROS-induced pathways in cancer to ultimately and selectively kill cancer cells by means of antimitotic pathways.

## Figures and Tables

**Table 1:** Percentage of MCF-7 cells occupying each cell cycle phase as determined by means of flow cytometry using propidium iodide after 24 h exposure. An asterisk (*) indicates *p*-value (*p* < 0.05) compared to ESE-one treated cells.

MCF-7 24 h Exposure				
	Sub-G_1_	G_1_	S-Phase	G_2_/M
Cells cultured in complete growth medium	1.82 ± 0.16	68.98 ± 0.12	11.8 ± 1.79	18.19 ± 0.59
Vehicle-treated cells	1.68 ± 0.13	63.7 ± 2.46	11.59 ± 1.71	22.59 ± 0.14
ESE-one only	17.23 ± 2.65	23.83 ± 0.83	11.61 ± 0.96	47.82 ± 3.44
Tiron − ESE-one	2.45 ± 0.65	74.16 ± 2.83	8.62 ± 1.14	14.13 ± 1.97
Tiron + ESE-one	13.47 ± 2.5	50.52 ± 3.64	12.97 ± 3.27	16.94 ± 1.13 *
Trolox − ESE-one	1.52 ± 0.40	62.83 ± 1.81	11.45 ± 1.23	24.44 ± 2.33
Trolox + ESE-one	23.68 ± 3.49	31.57 ± 3.30	13.13 ± 2.05	23.89 ± 3.84 *
DMTU − ESE-one	3.42 ± 1.02	69.09 ± 1.85	11.11 ± 1.33	17.68 ± 1.24
DMTU + ESE-one	10.65 ± 1.42	28.61 ± 1.13	12.0 ± 0.69	49.11 ± 0.82

**Table 2:** Percentage of MDA-MB-231 cells occupying each cell cycle phase as determined by means of flow cytometry using propidium iodide after 24 h exposure. An asterisk (*) indicates *p*-value (*p* < 0.05) compared to ESE-one treated cells.

MDA-MB-231 24 h Exposure				
	Sub-G_1_	G_1_	S-Phase	G_2_/M
Cells cultured in complete growth medium	0.50 ± 0.14	67.79 ± 0.23	11.45 ± 0.39	20.15 ± 0.52
Vehicle-treated cells	0.58 ± 0.11	68.10 ± 2.14	12.71 ± 2.20	18.51 ± 1.71
ESE-one only	25.72 ± 1.62	7.57 ± 1.33	8.18 ± 1.62	59.0. ± 4.62
Tiron − ESE-one	3.45 ± 0.88	71.57 ± 3.90	9.85 ± 1.45	13.42 ± 1.80
Tiron + ESE-one	15.21 ± 1.57 *	28.25 ± 2.44	17.50 ± 2.19	41.99 ± 1.76 *
Trolox − ESE-one	2.68 ± 0.87	71.76 ± 3.95	6.67 ± 0.87	22.13 ± 1.37
Trolox + ESE-one	27.48 ± 2.56	16.95 ± 1.29	6.94 ± 1.02	42.98 ± 1.69 *
DMTU − ESE-one	3.07 ± 1.11	62.76 ± 2.96	11.23 ± 1.05	21.60 ± 0.55
DMTU + ESE-one	15.73 ± 2.18 *	12.26 ± 0.81	8.35 ± 0.21	56.55 ± 4.21

**Table 3:** Percentage of MCF-7 cells occupying each cell cycle phase as determined by means of flow cytometry using propidium iodide after 48 h exposure. An asterisk (*) indicates *p*-value (*p* < 0.05) compared to ESE-one treated cells.

MCF-7 48 h Exposure				
	Sub-G_1_	G_1_	S-Phase	G_2_/M
Cells cultured in complete growth medium	1.31 ± 0.08	83.55 ± 2.14	5.68 ± 0.30	11.5 ± 1.68
Vehicle-treated cells	1.32 ± 0.11	84.69 ± 0.82	5.55 ± 0.67	8.73 ± 0.55
ESE-one only	65.30 ± 0.28	22.57 ± 0.86	4.18 ± 0.04	7.81 ± 0.59
Tiron − ESE-one	8.27 ± 0.11	83.77 ± 0.35	3.85 ± 0.42	3.58 ± 0.45
Tiron + ESE-one	28.27 ± 1.31 *	34.77 ± 1.65	12.08 ± 1.27	23.52 ± 1.51 *
Trolox − ESE-one	1.17 ± 0.01	67.94 ± 0.00	6.14 ± 0.52	20.76 ± 0.34
Trolox + ESE-one	54.80 ± 3.69 *	23.03 ± 0.65	8.66 ± 0.42	14.39 ± 2.48
DMTU − ESE-one	1.77 ± 0.01	87.05 ± 0.40	7.15 ± 0.52	5.47 ± 1.18
DMTU + ESE-one	46.81 ± 0.84 *	24.53 ± 0.98	5.63 ± 0.37	11.63 ± 1.97

**Table 4:** Percentage of MDA-MB-231 cells occupying each cell cycle phase as determined by means of flow cytometry using propidium iodide after 48 h exposure. An asterisk (*) indicates *p*-value (*p* < 0.05) compared to ESE-one treated cells.

MDA-MB-231 48 h Exposure				
	Sub-G_1_	G_1_	S-Phase	G_2_/M
Cells cultured in complete growth medium	0.80 ± 0.16	78.09 ± 0.07	6.32 ± 0.19	13.09 ± 0.75
Vehicle-treated cells	1.28 ± 0.28	76.34 ± 4.69	6.65 ± 2.21	11.96 ± 1.3
ESE-one only	52.1 ± 8.36	23.82 ± 0.39	5.76 ± 1.04	11.76 ± 0.67
Tiron − ESE-one	4.12 ± 1.55	80.66 ± 4.18	4.61 ± 0.87	4.55 ± 0.71
Tiron + ESE-one	29.4 ± 2.59 *	48.8 ± 8.24	5.92 ± 2.40	27.28 ± 5.71 *
Trolox − ESE-one	2.15 ± 0.69	80.71 ± 1.40	7.19 ± 1.42	10.59 ± 1.42
Trolox + ESE-one	39.51 ± 0.35 *	28.16 ± 0.22	10.28 ± 0.35	23.56 ± 3.16
DMTU − ESE-one	0.96 ± 0.08	79.96 ± 1.10	7.39 ± 1.92	13.13 ± 1.31
DMTU + ESE-one	32.48 ± 2.09 *	30.86 ± 0.04	10.28 ± 2.89	20.61 ± 0.16

**Table 5:** Percentage of MCF-7 cell polarity of the mitochondrial membrane potential as determined by means of flow cytometry. An asterisk (*) indicates *p*-value (*p* < 0.05) compared to ESE-one treated cells.

MCF-7 Cells		
	Polarized	Depolarized
Cells cultured in complete growth medium	97.2 ± 1.7	2.6 ± 1.5
Vehicle-treated cells	97.8 ± 1.1	2.2 ± 1.1
ESE-one only	85.4 ± 0.2	14.6 ± 0.2
CCCP	67.0 ± 1.2 *	33.0 ± 1.2 *
Tiron − ESE-one	93.7 ± 1.0	6.0 ± 1.0
Tiron + ESE-one	90.6 ± 1.0 *	9.3 ± 1.0 *
Trolox − ESE-one	96.6 ± 1.0	3.3 ± 1.0
Trolox + ESE-one	80.7 ± 6.1	19.3 ± 6.1
DMTU − ESE-one	97.6 ± 1.2	2.4 ± 1.2
DMTU + ESE-one	84.2 ± 4.0	15.8 ± 4.1

**Table 6:** Percentage of MDA-MB-231 cell polarity of the mitochondrial membrane potential as determined by means of flow cytometry. An asterisk (*) indicates *p*-value (*p* < 0.05) compared to ESE-one treated cells.

MDA-MB-231 Cells		
	Polarized	Depolarized
Cells cultured in complete growth medium	96.7 ± 2.3	2.9 ± 1.9
Vehicle-treated cells	96.0 ± 1.7	3.8 ± 1.8
ESE-one only	75.8 ± 8.0	24.1 ± 8.1
CCCP	64.4 ± 16.8	35.6 ± 16.5
Tiron − ESE-one	97.1 ± 2.4	2.8 ± 2.5
Tiron + ESE-one	78.0 ± 3.4	21.6 ± 3.9
Trolox − ESE-one	93.7 ± 2.2	6.7 ± 3.1
Trolox + ESE-one	71.1 ± 4.5	28.8 ± 4.5
DMTU − ESE-one	71.6 ± 3.5	8.0 ± 3.2
DMTU + ESE-one	58.5 ± 1.3 *	41.4 ± 1.5 *

## Data Availability

This article contains all relevant analyzed data obtained by this study. Corresponding author may be contacted for all original raw data.

## Sample Availability

Samples of compounds are not available from the authors.
